# Association of household net worth with healthcare costs after radical cystectomy using real‐world data

**DOI:** 10.1002/cam4.7116

**Published:** 2024-03-30

**Authors:** Samuel L. Washington, Peter E. Lonergan, Anobel Y. Odisho, Maxwell V. Meng, Sima P. Porten

**Affiliations:** ^1^ Department of Urology, Helen Diller Family Comprehensive Cancer Center University of California, San Francisco San Francisco California USA; ^2^ Department of Epidemiology and Biostatistics University of California, San Francisco San Francisco California USA; ^3^ Department of Urology St. James's Hospital Dublin Ireland; ^4^ Department of Surgery Trinity College Dublin Ireland

**Keywords:** bladder cancer care, healthcare utilization, household net worth

## Abstract

**Background:**

Financial toxicity of bladder cancer care may influence how patients utilize healthcare resources, from emergency department (ED) encounters to office visits. We aim to examine whether greater household net worth (HHNW) confers differential access to healthcare resources after radical cystectomy (RC).

**Methods:**

This population‐based cohort study examined the association between HHNW and healthcare utilization costs in the 90 days post‐RC in commercially insured patients with bladder cancer. Costs accrued from the index hospitalization to 90 days after including health plan costs (HPC) and out‐of‐pocket costs (OPC). Multivariable logistic regression models were generated by encounter (acute inpatient, ED, outpatient, and office visit).

**Results:**

A total of 141,903 patients were identified with HHNW categories near evenly distributed. Acute inpatient encounters incurred the greatest HPC and OPC. Office visits conferred the lowest HPC while ED visits had the lowest OPC. Black patients harbored increased odds of an acute inpatient encounter (OR 1.22, 95% CI 1.16–1.29) and ED encounter (OR 1.20, 95% CI 1.14–1.27) while Asian (OR 0.76, 95% CI 0.69–0.85) and Hispanic (OR 0.74, 95% CI 0.69–0.78, *p* < 0.001) patients had lower odds of an outpatient encounter, compared to White counterpart. Increasing HHNW was associated with decreasing odds of acute inpatient or ED encounters and greater odds of office visits.

**Conclusions:**

Lower HHNW conferred greater risk of costly inpatient encounters while greater HHNW had greater odds of less costly office visits, illustrating how financial flexibility fosters differences in healthcare utilization and lower costs. HHNW may serve as a proxy for financial flexibility and risk of financial hardship than income alone.

## Abstract 2

Our study of 141,903 patients, representing the largest cohort of commercially insured patients to examine the association between household net worth (HHNW) and healthcare utilization costs after RC to date, showed that lower HHNW conferred greater risk of acute inpatient encounters (and higher costs) while greater HHNW had greater odds of office visits (and lower costs). Greater financial flexibility fosters differences in healthcare utilization (and lower costs). HHNW may provide a more comprehensive measure of financial flexibility than income alone and serve as a proxy for healthcare access.

## 1 INTRODUCTION

In 2022, an estimated 81,180 new cases of bladder cancer will be diagnosed in the Unites States with approximately 20% being muscle‐invasive.^1^ Radical cystectomy (RC) remains the gold standard for the treatment of muscle‐invasive bladder cancer, yet incurs the risk of financial toxicity or hardship with significant costs exceeding $69,000 healthcare dollars per individual.^2^, ^3^ Household income, one of the most commonly‐used proxies for socioeconomic status, refers to household total cash income in the 12 months prior to study inclusion without assessments of additional assets and existing debt. However, income imperfectly measures household net worth (HHNW), a more comprehensive estimate of one's net economic standing (i.e., value of financial assets minus liabilities). This inaccuracy increases for retired or elderly individuals for whom household income may not truly represent their overall financial resources including stocks, economic reserves, and accumulated privilege.^2^ Furthermore, greater net worth more commonly clusters in counties or regions with greater household income and greater healthcare resources—effectively serving as a proxy not only for financial flexibility but also greater quality of care.^3^ Few studies have assessed how a patient's economic standing, rather than income alone, relates to healthcare resource utilization for those with bladder cancer. As a result, our understanding of the extent of financial toxicity and its impact on patient behavior remains incomplete.

To date, it is not known whether greater HHNW affords more cost‐effective care through greater access to and utilization of healthcare resources (e.g., outpatient follow‐up which may avert hospitalizations and/or emergency department [ED] encounters) or, inversely, lower socioeconomic status predicts underutilization of healthcare resources. We hypothesize that net worth is independently associated with variable methods of healthcare utilization after RC. Therefore, we aim to examine the association between HHNW, variations in healthcare utilization, and costs within 90 days after RC using comprehensive real‐world data in a commercially insured population of patients with bladder cancer.

## 2 METHODS

### 2.1 Study population

The OptumLabs® Data Warehouse (OLDW) is a unique database with over 200 million enrollees, nearly two‐thirds of the population of the United States. It includes de‐identified medical and pharmacy claims, laboratory results, and enrollment records for commercial and Medicare advantage enrollees. The OLDW contains longitudinal health information on enrollees and patients, representing a mixture of ages and geographical regions across the United States. Cohort selection was conducted through sequential structured queries within the OptumLabs® database. We identified a combination of ICD‐9 and ICD‐10 codes to identify Black, White, Asian, and Hispanic patients diagnosed with bladder cancer between January 1, 2007 and April 10, 2021 who underwent RC using a combination of ICD‐9 and ICD‐10 codes (bladder cancer, ICD‐9188.0‐9, 233.7; ICD‐10 67.0‐9, D09; RC, ICD‐9 57.7; ICD‐10 Z90.6) All patients had continuous insurance enrollment of at least 6 months prior to the index date (defined as date of hospitalization for RC) and 6 months after the index date. Patients admitted to a long‐term care facility (*n* = 264) were excluded. All claims data and associated costs from the index date to 90 days after the index date were abstracted directly from the database. The study was deemed minimal risk by the local institutional review board review due to the use of a deidentified data and thus exempt from further review. The last date of data abstraction was April 10, 2021.

### 2.2 Independent variables

Demographic data on age, race/ethnicity, gender, education, and household income were abstracted with additional data on homeownership status, HHNW, health plan costs (HPC), and out‐of‐pocket costs (OPCs). Age at index hospitalization was determined by calculating the total number of days lived from the year of birth to July 1 of the year of hospitalization. The midpoint of the year (July 1) was used as it would average out differences. The total number of days lived was then divided by 365.25 to calculate the age at index date. Race/ethnicity was reported using four categories: non‐Hispanic Black reported as Black, non‐Hispanic White reported as White, Hispanic, Asian. Further disaggregation could not be performed due to limited data availability. Gender was reported as male or female. Education was based on data from the U.S Census Bureau's American Community Survey and grouped as follows: <12th grade, high school (HS) diploma, <Bachelors, and at least a Bachelor's degree (Bachelors+). Household income was categorized as follows: <$40,000; 40,000–74,999; 75,000–124,9999; 125,000–199,999; >200k; and Unknown/Not reported.

Homeownership status was reported as probable homeowner, probable renter, or unknown/not reported. Homeownership status was obtained from property deeds, property tax assessments, and other publicly available information from over 98% of all US counties. Occupation type was available for 10%–27% of records and thus excluded. For HHNW, a “household” was defined as all individuals with the same surname living on the same street address. All individuals within the same household have the same estimated HHNW. HHNW was estimated using household assets and liabilities (net worth = assets—liabilities such as income, credit card statements, loan amounts, and loan payments) data from public and private consumer data for each household.

### 2.3 Outcome measures

Healthcare encounters were identified using mutually exclusive Place of Service codes from the Centers for Medicare and Medicaid Services occurring within the study period. Encounters were classified as acute inpatient, ED, outpatient, and office visit. Encounters in offices, military treatment facilities, federally qualified health centers, community mental health centers, public health clinics, and rural health clinics were categorized as office visits; visits to on campus‐outpatient hospitals, ambulatory surgical centers, outpatient rehabilitation facilities, and end‐stage renal disease treatment facilities were categorized as outpatient visits. Data on length of stay (sum of days from admit to discharge), number of unique encounters, and costs was abstracted. Cost categories included HPC and OPC within the study period. HPC were defined as the sum of all health plan paid cost for claims associated with each encounter revenue code. OPC were calculated as the sum of all OPCs for claims, that is, the costs paid by the patient.

### 2.4 Statistical analysis

Descriptive statistics were generated to describe the demographic, socioeconomic status, and cost characteristics of the study cohort. Means and standard deviations (SD) are reported for continuous variables. Frequencies are reported for categorical variables. Summary statistics were stratified by each encounter type to identify factors that associated with the encounter of interest. Separate multivariable logistic regression models were then generated for each encounter type (acute inpatient, ED, outpatient, and office visit). Explanatory variables included age, race/ethnicity, gender, education, and HHNW. HHNW includes household income and thus household income was excluded from the multivariable regression models. Odds ratios (OR) are presented with 95% confidence intervals (CI) and *p*‐values. A *p* value of <0.05 was deemed statistically significant. Statistical analyses were performed using RStudio (V1.1.456, © 2009–2018 RStudio, Inc.).

## 3 RESULTS

### 3.1 Overall cohort

The study cohort was comprised of 141,903 commercially insured individuals who underwent RC for bladder cancer. Mean age at index date was 69.5 years (SD 10.7) with a male predominance (74.4%). Table 1 describes the characteristics of the overall cohort. Of the individuals identified, 2.4% were Black, 11.4% Hispanic, and 8.1% Asian. The majority (62.5%) had less than a college education. HHNW categories were near evenly distributed amongst the study cohort. A total of 186,459 acute inpatient encounters, 187,184 ED encounters, 352,127 outpatient encounters, and 356,921 office visits occurred during the study period.

**TABLE 1:** Demographics for entire study cohort (*n* = 141,903).

Variable	*n* (%)
Age, mean (SD)	69.5 (10.7)
Race	
African American/Black	3349 (2.4)
White	110,840 (78.1)
Hispanic	16,177 (11.4)
Asian	11,537 (8.1)
Gender	
Male	105,577 (74.4)
Female	36,326 (25.6)
Education	
<12th grade	412 (0.3)
HS diploma	31,531 (22.2)
<Bachelors	56,790 (40.0)
Bachelors+	15,737 (11.1)
Unknown/not reported	37,833 (26.4)
Household income (dollars)	
<40,000	23,755 (16.7)
40,000–74,999	24,634 (17.4)
75,000–124,9999	22,165 (15.6)
125,000–199,999	9224 (6.5)
>200k	5023 (3.5)
Household net worth (dollars)	
<25,000	19,256 (13.6)
25,000–149,000	19,483 (13.7)
150,000–249,000	11,792 (8.3)
250,000–499,000	18,462 (13.0)
>500,000	22,818 (16.1)
Unknown/not reported	13,041 (9.2)
Health plan costs (dollars per person)	
Mean (SD)	28069.90 (83766.65)
Median (IQR)	5679.39 (1403.71–26334.55)
Out‐of‐pocket costs (dollars per person)	
Mean (SD)	1927.07 (3296.50)
Median (IQR)	698.31 (135.69–2613.14)

Abbreviations: HS, high school; IQR, interquartile range; SD, standard deviation.

### 3.2 Healthcare encounters and costs

In the 90‐day period after RC, 45.2% reported an acute inpatient, 20.2% ED, 85.1% outpatient, and 85.3% an office visit encounter. Nearly one‐third (31.7%) had both an ED and acute inpatient encounter while 40% had both ED and outpatient encounters. Nearly half (45%) had outpatient encounters without an ED visit while 5.3% had an ED visit without subsequent follow‐up. 9.6% had no follow‐up encounters reported within 90 days of RC.

Acute inpatient encounters incurred the greatest HPC (mean $24,642.80, SD $57,218.41) and OPC (mean $1428.24, SD $2108, Table 2). Office visits had the lowest HPC (mean $1126.78, SD $4119.12) costs while ED visits had the lowest OPC (mean $181.88; SD $399.65).

**TABLE 2:** Breakdown of estimated costs per healthcare encounter, stratified healthcare encounter type (*n* = number of encounters)^a^.

Variable	Acute inpatient (*n* = 186,459 encounters)	ED (*n* = 187,184 encounters)	Outpatient (*n* = 352,127 encounters)	Office visit (*n* = 356,921 encounters)
# Events/person				
Mean (SD)	1.24 (0.61)	1.44 (1.00)	7.19 (7.86)	5.76 (5.58)
Median (IQR)	1 (1–1)	1 (1–2)	5 (3–9)	4 (2–7)
Length of stay				
Mean (SD)	8.97 (11.93)	–	–	–
Median (IQR)	6 (4–10)	–	–	–
Health plan costs (in dollars)				
Mean (SD)	24,642.80 (57,218.41)	1511.11 (3130.40)	4269.17 (11,472.94)	1126.78 (4119.12)
Median (IQR)	11,326.26 (3792.80–24,730.41)	536.14 (195.55–1546.27)	1167.76 (215.88–4089.99)	315.38 (107.32–810.25)
Out‐of‐pocket costs (in dollars)				
Mean (SD)	1428.24 (2018)	163.66 (451.03)	439.99 (1009.03)	181.88 (399.65)
Median (IQR)	926.38 (113.43–2029.62)	22.11 (0–138.07)	81.77 (0–484.30)	82.84 (26.84–193.80)

Abbreviations: ED, emergency department; IQR, interquartile range; SD, standard deviation.

^a^ Patients may experience more than one of each encounter and more than one encounter type.

### 3.3 Acute inpatient encounters

Approximately 45.2% of the cohort experienced an acute inpatient encounter, with a median length of stay of 6 days (IQR, 4–10). On multivariable analysis for factors associated with an acute inpatient encounter (Table 3), Black individuals compared to White counterparts (OR 1.22, 95% CI 1.16–1.29, *p* < 0.001) and lower educational ascertainment compared to Bachelors degree (less than Bachelors, OR 1.05, 95% CI 1.00–1.10, *p* = 0.04; HS diploma, OR 1.17, 95% CI 1.11–1.23, *p* < 0.001) were associated with increased odds of an acute inpatient encounter after adjustments for age and gender. Increasing HHNW was associated with decreasing odds of an acute inpatient encounter (<$25,000, OR 0.89, 95% CI 0.83–0.95; $25,000‐149,000, OR 0.90, 95% CI 0.85–0.9; $150,000–249,000, OR 0.88, 95% CI 0.82–0.94; $250,000–499,000, OR 0.84, 95% CI 0.80–0.90; >$500,000, OR 0.72, 95% CI 0.68–0.76; compared to those without reported net worth, *p* < 0.001 for all).

**TABLE 3:** Multivariable logistic regression models for factors associated with each healthcare encounter (*n* = number of encounters)^a^

Variable	Acute inpatient (*n* = 186,459 encounters)			ED (*n* = 187,184 encounters)			Outpatient (*n* = 352,127 encounters)			Office visit (*n* = 356,921 encounters)		
	OR	95% CI	*p*	OR	95% CI	*p*	OR	95% CI	*p*	OR	95% CI	*p*
Age (years)												
Race	0.08	0.07–0.09	<0.001	1.02	1.02–1.02	<0.001	0.99	0.98–0.99	<0.001	0.97	0.96–0.97	<0.001
White	Ref.	Ref.	Ref.	Ref.	Ref.	Ref.	Ref.	Ref.	Ref.	Ref.	Ref.	Ref.
Asian	0.93	0.83–1.05	0.25	0.86	0.76–0.97	0.02	0.76	0.69–0.85	<0.001	0.81	0.73–0.90	<0.001
Black	1.22	1.16–1.29	<0.001	1.20	1.14–1.27	<0.001	0.95	0.90–1.00	0.06	0.92	0.87–0.97	0.002
Hispanic	1.04	0.97–1.11	0.25	1.04	0.97–1.11	0.30	0.74	0.69–0.78	<0.001	0.82	0.77–0.88	<0.001
Gender												
Male	Ref.	Ref.	Ref.	Ref.	Ref.	Ref.	Ref.	Ref.	Ref.	Ref.	Ref.	Ref.
Female	0.97	0.94–1.01	0.09	0.90	0.87–0.03	<0.001	0.95	0.92–0.98	0.002	0.96	0.93–0.99	0.03
Education												
Bachelors	Ref.	Ref.	Ref.	Ref.	Ref.	Ref.	Ref.	Ref.	Ref.	Ref.	Ref.	Ref.
Less than bachelors	1.05	1.00–1.10	0.04	1.09	1.04–1.14	0.001	1.05	1.00–1.10	0.03	1.03	0.98–1.07	0.25
High school diploma	1.17	1.11–1.23	<0.001	1.16	1.10–1.23	<0.001	1.08	1.02–1.13	0.007	1.05	1.0–1.1	0.06
Less than 12th grade	1.06	0.75–1.47	0.74	1.15	0.82–1.57	0.4	0.72	0.54–0.97	0.03	0.88	0.65–1.20	0.4
Net worth												
Not reported	Ref.	Ref.	Ref.	Ref.	Ref.	Ref.	Ref.	Ref.	Ref.	Ref.	Ref.	Ref.
<25,000	0.89	0.83–0.95	<0.001	0.96	0.91–1.03	0.3	0.81	0.76–0.87	<0.001	0.89	0.83–0.94	<0.001
25,000–149,000	0.90	0.85–0.96	<0.001	0.87	0.82–0.92	<0.001	0.87	0.82–0.93	<0.001	1.04	0.98–1.11	0.2
150,000–249,000	0.88	0.82–0.94	<0.001	0.82	0.77–0.88	<0.001	0.95	0.89–1.01	0.1	1.16	1.08–1.24	<0.001
250,000–499,000	0.84	0.80–0.90	<0.001	0.76	0.72–0.81	<0.001	0.95	0.89–1.01	0.1	1.26	1.19–1.34	<0.001
>500,000	0.72	0.68–0.76	<0.001	0.66	0.63–0.70	<0.001	0.87	0.82–0.92	<0.001	1.25	1.18–1.32	<0.001

^a^ Patients may experience more than one of each encounter and more than one encounter type.

### 3.4 Emergency department encounters

Black individuals were at greater odds (OR 1.20, 95% CI 1.14–1.27, *p* < 0.001) of an ED encounter while Asian individuals (OR 0.86, 95% CI 0.76–0.97, *p* = 0.02) had lower odds compared to White counterparts (Table 3). Women (OR 0.90, 95% CI 0.87–0.93, *p* < 0.001) had lower odds after adjustments compared to men. Increasing HHNW was associated with decreasing odds of an ED encounter ($25,000–149,000, OR 0.87, 95% CI 0.82–0.92; $150,000–249,000, OR 0.82, 95% CI 0.77–0.88; $250,000–499,000, OR 0.76, 95% CI 0.72–0.81; >$500,000, OR 0.66, 95% CI 0.63–0.70; compared to those without reported net worth, *p* < 0.001 for all).

### 3.5 Outpatient encounters

On multivariable analysis, Asian (OR 0.76, 95% CI 0.69–0.85, *p* < 0.001) and Hispanic (OR 0.74, 95% CI 0.69–0.78, *p* < 0.001) individuals had lower odds compared to White counterparts (Table 3). Odds for Black individuals did not differ significantly compared to White counterparts. Those with less than 12th grade education were less likely to have an outpatient encounter compared to those with at least a bachelor's degree (OR 0.72%, 95% CI 0.54–0.97, *p* = 0.03). Those with HHNW less than $25,000 (OR 0.81, 95% CI 0.76–0.87, *p* < 0.001) and greater than $500,000 (OR 0.87, 95% CI 0.82–0.93, *p* < 0.001) were associated with lower odds of an outpatient encounter compared to those without reported HHNW.

### 3.6 Office visit encounters

When comparing by office visit encounters, individuals with HHNW greater than $500,000 were more common amongst those who had an office visit compared to those who did not (22.3% vs. 20.6%) and less common for those with HHNW less than $250,000 (10% vs. 12.3%, *p* < 0.001). Compared to white counterparts, all race/ethnicity groups had lower odds of an office visit encounter (*p* < 0.001 for all). Increasing HHNW was associated with increasing odds of an office visit (*p* < 0.001 for all) after adjustments.

## 4 DISCUSSION

In this cohort of 141,903 commercially insured individuals with bladder cancer who underwent RC, we found that nearly two‐thirds had an acute inpatient encounter (45.2%) or ED visit (20.2%) and the majority (85.3%) had at least one office visit in the 90 days after surgery. As expected, ED visits conferred the lowest OPC and office visits the lowest HPC. Our study shows that the association between greater HHNW confers differences in healthcare utilization (and lower healthcare costs), even within a cohort of individuals with commercial insurance. Our study represents one of the first, and largest, studies to leverage real‐world data to examine the relationship between HHNW and healthcare utilization within the first 90 days after RC.

Using real‐world data we observed variations in both HPC and OPC across the four types of healthcare encounters that may illustrate potential patterns of cost‐avoidance behaviors for individuals interacting with the healthcare system after RC. Not surprisingly, acute inpatient encounters incurred the greatest HPC, ED encounters the lowest OPC, and office visits the lowest HPC after cystectomy. Those undergoing RC bear both high risk of morbidity (30%–40% risk of readmission) as well as significant costs which may exceed $69,000 healthcare dollars for the individual from the surgical intervention.^4^, ^5^, ^6^ As a result, the risk of financial toxicity associated with RC remains high in magnitude relative to financial resources available. Several studies have examined the impact of financial toxicity on individuals with bladder cancer. A recent cross‐sectional survey‐based study of 226 patients with bladder cancer reported greater financial toxicity noted with higher OPC for cancer therapy, lower income, greater percentage loss of income.^7^ On multivariable analysis, income, employment status, and insurance were all significantly associated with financial toxicity. Although only 15% of patient underwent RC, this study provided important insight into the financial burden that individuals with bladder cancer face and which factors may mitigate the risk. Using a hospital‐based cohort of 994 cancer survivors of breast, lung, colorectal, or prostate cancer, one study showed that those experiencing financial hardships such as decreased income, borrowing money, cancer‐related debt, and accessing assets to pay for cancer care were 4.4 (95% CI 2.9–6.6) times more likely to skip doses of prescribed medication, refuse treatment, or not consult a physician when needed due to cost.^8^ Similar behaviors have been observed in individuals undergoing treatment for bladder cancer as well. A separate cross‐sectional survey‐based study of 138 patients with bladder cancer treated at a single academic institution noted that those with private insurance (22%) were least likely to report financial toxicity.^9^ Those who endorsed financial toxicity were more likely to report delays in care (*p* = 0.07) and attribute it to finance‐related factors such as inability to take time off work (*p* = 0.04) and inability to afford general expenses (*p* = 0.04) Conversely, older age (OR = 0.29, 95% CI 0.13, 0.65) significantly associated with lower odds of financial toxicity, a finding which alludes to how differences in wealth or net worth by age may mitigate the risk of financial toxicity. In this study we found that those with the greatest HHNW ($ >500,000) had greatest odds of office visits (OR 1.25, 95% CI 1.18–1.32) and lowest odds of an acute inpatient encounter (OR 0.72, 95% CI 0.68–0.76) compared to those who did not report HHNW, highlighting likely differences in utilization that also resulted in lower healthcare costs.

In this study office visits conferred the lowest HPC relative to all other types of healthcare encounters, highlighting a potential strategy for systems‐level cost‐reduction if implemented broadly. One such study sought to identify a model which would optimize the probability of detecting patients at risk of readmission using office visits and telephone calls by leveraging time‐delay analyses with data from the Healthcare Cost and Utilization Project State Inpatient Databases.^10^ The authors ultimately concluded that increasing outpatient contact via phone calls and office visits may decrease readmissions due to early detection of problems, reaching up to 36% of potential readmissions being prevented with an office visit and 4 telephone calls. Although this paradigm can optimize outpatient follow‐up to avoid readmissions and reduce avoidable healthcare expenditure, disparities in care due to differences by the social construct of race, insurance coverage, and financial hardship may hamper implementation. The interactions between race and financial hardship for individuals with bladder cancer mirrors those seen across medicine, with up to 50.3% of Black cancer survivors endorsing financial hardship due to debt or decreased income while White counterparts more commonly reported the ability to access assets such as refinancing/selling a home or using retirement funds to pay for care.^8^, ^11^ These findings represent a more comprehensive characterization of financial hardship and financial flexibility that the more common approach of relying on household income, insurance status, educational ascertainment, or the combination as the primary measures of socioeconomic status will not capture comprehensively. In this study, both race and HHNW were independently associated with the odds of an office visit within 90 days after RC. We found that Black individuals had 22% greater odds of an acute inpatient admission and 20% greater odds of an ED visit compared to white counterparts. All non‐white patients had 8%–19% lower odds of office visits compared to white counterparts, even within a cohort of commercially insured patients, and increasing HHNW was inversely associated with the odds of an acute inpatient encounter and congruent with the odds of an outpatient encounter. Our findings point to the need for more granular examinations of the interplay between wealth, race, and health. In order to better understand how financial hardship impacts care, the inclusion of more comprehensive assessments of financial resources that incorporate estimates of assets and debt—such as HHNW—are required for more accurate estimates of one's ability to manage cancer‐related healthcare expenses.

This study has limitations which need to be acknowledged. Data on clinico‐demographic factors commonly reported in cancer databases such as tumor characteristics, cancer staging, geographic location, and complications were not available in order to access more comprehensive socioeconomic measures and real‐word data for a large, national cohort of individuals with bladder cancer. For example, HHNW was available at the expense of data on unmeasured variations in regional differences in clinical practice, socioeconomic factors, direct measurements of availability of healthcare/specialty resources, indications for healthcare visits such as routine visits or for management of complications, and fragmentation of care. HHNW remains a variable that has not been explored fully, either in the clinical setting or in the literature, and thus its overall utility remains largely uncharacterized. This data restriction impairs our ability to understand the granular interactions between race, geography, and care that could further be explored with a larger, more diverse cohort. Like many other large population level datasets, the preassigned definition of HHNW limits our ability to assess whether households include persons of different surnames and may serve as an incomplete measure of the complexity of the true definition of “household” and net worth at the individual level. As the available data for both household income and HHNW are provided as categorical variables, rather than numerical values, we are limited in our ability to transform the data further due to the characteristics of the variables within the dataset.” Our study was unable to directly measure the quality of care received although detailed estimates of 90‐day costs, rather than the more commonly used 30‐day interval were available. Although this study allowed for linkage of associated encounter costs for each patient, this does not completely eliminate the risk of over‐ascertainment of cystectomy events which could be a source of confounding. This study does not have detailed data on differences and associated copays across healthcare plans, although these are likely mediators through which financial resources and economic standing (measured by net worth) influence healthcare utilization. In addition, this dataset does not allow for further exploration of individual‐level factors related to educational ascertainment and its influence on how individuals navigate the healthcare system. Lastly, we are unable to accurately characterize the experience and costs at the individual‐level due to the high number of combinations of healthcare encounters experienced within the study period. Our findings may have limited generalizability to specific individuals, although this limitation is inherent to all cohort studies. In the context of these limitations, this study represents the first and largest to utilize real‐world data to assess the relationship between HHNW, a comprehensive measure of financial resources, and healthcare utilization within 90 days of RC for a commercially insured cohort of individuals with bladder cancer.

## 5 CONCLUSION

Our study of 141,903 patients, representing the largest cohort of commercially insured patients to examine the association between HHNW and healthcare utilization costs after RC to date, showed that lower HHNW conferred greater risk of acute inpatient encounters (and higher costs) while greater HHNW had greater odds of office visits (and lower costs). Greater financial flexibility fosters differences in healthcare utilization (and lower costs). HHNW may provide a more comprehensive measure of financial flexibility than income alone and serve as a proxy for healthcare access.

## AUTHOR CONTRIBUTIONS

**Samuel L. Washington III:** Conceptualization (lead); data curation (lead); formal analysis (lead); investigation (lead); methodology (lead); project administration (lead); software (equal); writing – original draft (lead). **Peter E. Lonergan:** Conceptualization (supporting); investigation (supporting); writing – review and editing (supporting). **Anobel Y. Odisho:** Formal analysis (supporting); investigation (supporting); methodology (supporting); writing – review and editing (supporting). **Maxwell V. Meng:** Conceptualization (equal); investigation (equal); methodology (equal); supervision (equal); writing – review and editing (equal). **Sima P. Porten:** Conceptualization (equal); investigation (equal); methodology (equal); supervision (equal); writing – review and editing (equal).

## FUNDING INFORMATION

None.

## Data Availability

The data that support the findings of this study are available from OptumLabs. Restrictions apply to the availability of these data, which were used under license for this study. Data are available [Samuel L. Washington, Sima P. Porten] with the permission of OptumLabs.
